# Fabrication of Ciprofloxacin-Loaded Sodium Alginate Nanobeads Coated with Thiol-Anchored Chitosan Using B-390 Encapsulator Following Optimization by DoE

**DOI:** 10.3390/pharmaceutics16060691

**Published:** 2024-05-21

**Authors:** Mahwash Mukhtar, Ildikó Csóka, Josipa Martinović, Gordana Šelo, Ana Bucić-Kojić, László Orosz, Dóra Paróczai, Katalin Burian, Rita Ambrus

**Affiliations:** 1 Institute of Pharmaceutical Technology and Regulatory Affairs, Faculty of Pharmacy, University of Szeged, Eötvös u.6, 6720 Szeged, Hungary; mahwash.mukhtar@szte.hu (M.M.); csoka.ildiko@szte.hu (I.C.); 2 Faculty of Food Technology Osijek, Josip Juraj Strossmayer University of Osijek, F. Kuhača 18, 31 000 Osijek, Croatia; jgrgic2@ptfos.hr (J.M.); abucic@ptfos.hr (A.B.-K.); 3 Department of Medical Microbiology, Faculty of Medicine, University of Szeged, Dóm Square 10, 6720 Szeged, Hungary; orosz.laszlo@med.u-szeged.hu (L.O.); paroczai.dora@med.u-szeged.hu (D.P.); burian.katalin@med.u-szeged.hu (K.B.)

**Keywords:** anti-inflammatory, B-390 encapsulator, ionic gelation, nanobeads, sodium alginate, thiolated chitosan

## Abstract

Most infectious diseases of the gastrointestinal tract can easily be treated by exploiting the already available antibiotics with the change in administration approach and delivery system. Ciprofloxacin (CIP) is used as a drug of choice for many bacterial infections; however, long-term therapy and off-site drug accumulation lead to an increased risk of tendinitis and peripheral neuropathy. To overcome this issue, nanotechnology is being exploited to encapsulate antibiotics within polymeric structures, which not only facilitates dose maintenance at the infection site but also limits off-site side effects. Here, sodium alginate (SA) and thiol-anchored chitosan (TC) were used to encapsulate CIP via a calcium chloride (CaCl_2_) cross-linker. For this purpose, the B-390 encapsulator was employed in the preparation of nanobeads using a simple technique. The hydrogel-like sample was then freeze-dried, using trehalose or mannitol as a lyoprotectant, to obtain a fine dry powder. Design of Experiment (DoE) was utilized to optimize the nanobead production, in which the influence of different independent variables was studied for their outcome on the polydispersity index (PDI), particle size, zeta potential, and percentage encapsulation efficiency (% EE). In vitro dissolution studies were performed in simulated saliva fluid, simulated gastric fluid, and simulated intestinal fluid. Antibacterial and anti-inflammatory studies were also performed along with cytotoxicity profiling. By and large, the study presented positive outcomes, proving the advantage of using nanotechnology in fabricating new delivery approaches using already available antibiotics.

## 1. Introduction

Bacterial infections account for the highest number of infections globally, with a significant share in the global disease burden [1]. For decades, antimicrobial therapy using antibiotics has been the most widely used treatment option. However, frequent administration, misuse, and long-term therapy have led to antimicrobial resistance (AMR). Unfortunately, this is becoming a nightmare in the case of hospital- and community-acquired infections [2]. It is estimated that over 230 million people will be affected due to AMR, and the mortality rate will rise to 10 million people by the end of 2050 [3].

The goal of pharmaceutical research and development is to provide the community with safe and effective drug delivery systems to treat diseases. Over the past years, the oral route of administration has been and is still preferable to a majority of the population. However, this route has limitations in terms of low or incomplete absorption, hepatic first-pass effect, low bioavailability, reduced residence time, and high degradation or metabolism of drugs [4]. Nonetheless, the anatomical features of the gastrointestinal tract dispense a broad range of drug delivery approaches to be used. The glycoprotein mucin in the gastrointestinal tract provides not only the characteristics of protection but also the adhesiveness that can be further explored by developing a delivery system with high adhesion to mucin that can further improve the mean residence time of the therapy [5]. Many synthetic and natural polymers have features that can improve the efficiency of drug delivery systems. Here, for this purpose, polymers such as sodium alginate (SA) and a derivative of chitosan (CS) were used. SA is an anionic linear polysaccharide, composed of blocks of α-1,4-l-guluronic acid (G) and β-1,4-d-manurunic acid (M) units, and is derived from brown seaweed [6]. SA was selected because of its unique sol–gel and mucoadhesive properties, along with its ability to develop cross-linking. Interestingly, SA has also shown a protective effect in the inflamed colon model by inhibiting pro-inflammatory cytokines [7]. Likewise, CS is another biocompatible and biodegradable polymer derived from marine sources comprising poly Β-(1-4)-2-amino-2-deoxy-d-glucose units. CS also manifests antibacterial properties, as this cationic polymer can destabilize bacterial replication by binding to the bacterial cell wall [8,9,10]. However, the polymer CS has been widely explored in conjugation with SA; therefore, we developed the derivative of CS, thiolated chitosan (TC), to further enhance the antibacterial and mucoadhesive features [11,12].

The high surface-to-volume ratio of nanoparticles and their high drug-loading capacity make them a highly versatile drug delivery vehicle. Moreover, the use of a polymeric nanosystem can be an effective approach for targeting gastrointestinal tract infections, owing to its biodegradation, improved residence time, high epithelial membrane permeability, high bioavailability, protection from acidic gastric media, and high absorption in the intestine [13]. The small size of the particles also makes them ideal candidates for passive targeting in colon infections by reaching out to the inflamed parts by infiltration.

Hence, we explored the potential of a nanoparticulate drug delivery system in this work by encapsulating antibiotic ciprofloxacin (CIP), which is the most commonly administered drug in community- and hospital-acquired infections. Here, we used the B-390 encapsulator to load the drug in the polymeric cores [14]. The synthesis approach was based on a simple ionic gelation technique that involved the use of a cross-linker to develop a matrix-like system that looked like nanobeads in suspension form. Moreover, the statistically robust Design of Experiment (DoE) method was used to optimize the samples by studying the various influencing factors with a limited number of runs on the outcomes.

## 2. Materials and Methods

### 2.1. Materials

Ciprofloxacin base (CIP; Mw = 331.35) was gifted by Teva Pharmaceutical Works Ltd. (Debrecen, Hungary). Chitosan (CS) (75–85% deacetylated, low molecular weight, 50–190 kDa, poly(D-glucosamine)), calcium chloride, dialysis membrane, high retention (12,000–14,000 Mw cut-off), trehalose, alginic acid sodium salt from brown algae (low viscosity; 4–12 cP, 1% in H_2_O (25 °C)), minimal essential medium (MEM) with Earle’s salts, and TRI reagent were obtained from Sigma (St. Louis, MO, USA). Sodium dodecyl sulfate (SDS) and 3-(4,5-dimethylthiazol-2-yl)-2,5-diphenyltetrazolium bromide (MTT) were obtained from Sigma-Aldrich (Chemie GmbH, Steinheim, Germany), and 1-ethyl-3-3(3-dimethylamino propyl) carbodiimide hydrochloride (EDAC) and thioglycolic acid (TGA) were ordered from Tokyo Chemical Industry Co., Ltd., (Tokyo, Japan). Glacial acetic acid (GAA) was purchased from Molar Chemicals Kft. (Halásztelek, Hungary). Mannitol (anhydrous β form) was purchased from MEGGLE Group, Wasserburg, Germany. All the chemicals were of high purity or reagent grade.

### 2.2. Bacterial Strains

The following strains were used in our experiments: *Escherichia coli* (ATCC^®^ 25922) and *Pseudomonas aeruginosa* (ATCC^®^ 27853).

### 2.3. Synthetic Procedure for Thiolated Chitosan

The polymer TC was synthesized using our previously reported method [15]. In short, 6.89 mL of TGA was added to the CS (1% solution in GAA), which led to the formation of an amide bond between the TGA and CS. Later, the carboxylic acid groups of TGA were activated by adding 50 mM 1-ethyl-3-3(3-dimethylamino propyl) carbodiimide hydrochloride (EDAC) to the mixture, followed by the mixing of hydroxylamine solution to prevent oxidation during the whole procedure. 1 M NaOH was used to adjust the pH of the polymeric suspension to 5.1. Finally, the unbound TGA was removed from the polymeric mixture using dialysis, in which the dialysis medium was changed every 6 h (5 L of 5 mM HCl solution, 5 L of 5 mM HCl for another 6 h, 5 L of 5 mM HCl with 1% NaCl for 6 h, 1 mM HCl solution, and 1 mM HCl again for 6 h at pH 4). Lyophilizer (Alpha 1–2 LD plus, Germany) was employed to lyophilize the mixture, which was frozen to −80 °C before lyophilization. An off-white dry mass was acquired, and it was stored at 4 °C [16].

### 2.4. Determination of Thiol Group Immobilization on CS and Disulfide Bonds

Ellman’s reagent was used to determine the immobilization of thiol groups on CS. The procedure involved the dissolution of TC in deionized water, adding 0.20 mL of phosphate buffer pH 8 and 0.5 mL of Ellman’s reagent. This TC mixture was incubated for 2.5 h at room temperature, followed by centrifugation at 20376 g. Acquired supernatant was quantified on the micro-titration plate reader (Perkin Elmer, Waltham, MA, USA) at 430 nm wavelength, and thiol groups immobilized on CS were evaluated against TGA standard [17].

Also, the determination of disulfide bonds helped in the quantification of free thiol groups on the polymer, for which TC was dissolved in distilled water with 650 µL of phosphate buffer (pH 6.8). After 20 min, sodium borohydride was added into the polymeric suspension, followed by incubation at room temperature for 1 h. Then, 5 M HCl was dropped into the incubated suspension to remove the leftover sodium borohydride. Ellman’s reagent (0.1 mL) along with phosphate buffer (pH 8.5) was then added to the mixture, which was left for incubation again for 1 h. The sample was then transferred to a 96-well plate, and absorbance was read using a microtitration plate reader (Perkin Elmer). Unattached thiol groups were subtracted from the total number of thiol groups attached to the polymer to quantify the disulfide bonds [18].

### 2.5. Design of Experiment Using the Plackett–Burman Design (PBD) Method

PBD was selected for the optimization of the samples. It is a robust screening design with a large number of factors and fewer experimental runs. It scrutinizes the most influential independent variables among a large number of selected variables and allows for the optimization of samples without tedious conventional hit-and-trial methods [19]. PBD is not an exhaustive model and identifies the main principal factors at only two levels. The major influencing parameters can therefore be studied by exploring ranges of parameter values. Moreover, it also reduces the data built up and gives the information required in an efficient manner. In this case, 7 independent variables were studied involving a total of 8 runs. Analysis of variance (ANOVA) was used for the statistical analysis of each model coefficient, with significance at the 95% level (*p* < 0.05). STATISTICA^®^ 12 software was employed to determine the impact of the independent variables with 2-level screening (Table 1) on the dependent variables (Y1: polydispersity index (PDI), Y2: particle size, Y3: zeta potential, Y4: % encapsulation efficiency (% EE)), with a total of 8 runs (Table 2). The established linear equation used for the PBD is

Y = β_0_ + β_1_ X_1_ + β_2_ X_2_ + β_3_ X_3_ + β_4_ X_4_ + β_5_ X_5_ + β_6_ X_6_ + β_7_ X_7_ (1)

where Y is the response, β_0_ is a constant, and β_1_, β_2_, β_3_, β_4_, β_5_, β_6_, and β_7_ are the coefficients of the factors X_1_, X_2_, X_3_, X_4_, X_5_, X_6_, and X_7_, respectively.

### 2.6. Preparation of Nanobeads via Ionic Gelation

Nanobeads were prepared by the simple ionic gelation method using encapsulation advice (Büchi B-390, Flawil, Switzerland) [20]. Briefly, a 1% and 1.5% SA polymeric mixture was prepared and stirred overnight. CIP at a concentration of 50 and 100 mg solubilized in a small volume of methanol was poured into the SA polymeric mixture. A TC polymeric mixture of 0.5% and 1% was also prepared in 0.05 M GAA, and the pH was adjusted to 4.9 using 0.1 N NaOH solution. CaCl_2_ was used as a cross-linker, which was solubilized in the TC suspension. For all experiments, 80 and 120 µm nozzles, an electrode voltage of 400 V, and a pressure of 250 mbar were used. In the next step, encapsulation was performed, in which the SA and CIP mixture was fed from the inlet tube into the TC and CaCl_2_ mixture. In the presence of CaCl_2_, the anionic carboxyl group of SA and the cationic amine group of TC cross-linked to form nanobeads, which were later filtered and washed with distilled water to remove the remnants of CaCl_2_. A hydrogel-like consistency was obtained, and the mixture was stirred for some time.

### 2.7. Freeze-Drying to Obtain Dry Powders

The nanobeads with a hydrogel-like consistency were freeze-dried using a freeze dryer (Alpha 2–4 LSCplus, Christ, Osterode am Harz, Germany) at −83 °C under a vacuum of 0.250 mbar for 24 to 72 h. Before freeze-drying, the samples were frozen at −80 °C in the freezer (SWUF Ultra Low Temperature Smart Freezer, Witeg, Wertheim, Germany).

### 2.8. Particle Size, Polydispersity Index (PDI), and Surface Charge

The measurement of particle size, PDI, and zeta potential was carried out using the Malvern zeta sizer Nano ZS (Malvern instrument, Malvern, Worcestershire, UK) equipped with a 633 nm wavelength laser based on quasi-elastic light scattering with a fixed scattering angle of 90 degrees at 25 °C. For sample evaluation, the lyophilized dry powders were redispersed in water, sonicated, and filtered using a 0.45 µm filter mesh to avoid particle aggregation. Later, the samples were filled into DTS1070, a disposable folded capillary cell. The integration time selected was 110 s for 11 runs as the automatic mode measurement duration. All experiments were performed in triplicate and are expressed as mean ± SD.

### 2.9. % Encapsulation Efficiency (% EE)

The % EE of the samples was evaluated by centrifugation of the samples at 10,062 g for 30 min. The supernatant was withdrawn and filtered before quantification of the drug analytically at 274 nm using a UV–visible spectrophotometer. Equation (2) was used to calculate % EE. The drug content (% *w*/*w*), also referred to as drug loading, was calculated as well using Equation (3), for which the sample powder was added to 0.05 M GAA (a common solvent for drug and polymers). After 4 h, a certain volume was withdrawn, filtered, and quantified at 274 nm using a UV–visible spectrophotometer. The calculated % drug content (*w*/*w*) has been mentioned in Supplementary Table S2.

% Encapsulation Efficiency = (Total drug − Free drug)/(Total drug) × 100(2)

% Drug content = (Mass of drug in sample)/(Mass of sample recovered) × 100(3)

### 2.10. Morphological Examination

The dry powders obtained were examined for their surface morphology using scanning electron microscopy (SEM) (Hitachi S4700, Hitachi Scientific Ltd., Tokyo, Japan) at 2.0–5.0 kV. A pressure of 1.3–13.0 mPa was maintained throughout the imaging. The samples were mounted on the sample disk and gold-sputtered before imaging.

### 2.11. Thermal and Structural Investigation

The thermal investigation of the samples was done using differential scanning calorimetry (Mettler Toledo DSC 821e, Mettler Inc., Schwerzenbach, Switzerland). For the analysis, 3–5 mg accurately weighed samples were hermetically sealed in aluminum pans. The samples were heated at a constant rate of 10 °C/min in the temperature range of 25 to 300 °C in an inert argon atmosphere (100 mL/min). STARe software version 9.3 was used for the evaluation of the thermograms.

The structural investigation was then presented using X-ray powder diffraction recorded with a BRUKER D8 Advance X-ray powder diffractometer (Bruker AXS GmbH, Karlsruhe, Germany), with Cu radiation at λ = 1.5406 Å over a 2θ range of 3–40° at a step time of 0.1 s. The scanning was done at 40 kV and 40 mA, and the VANTEC-1 detector was used to acquire diffractograms. DIFFRACTPLUS EVA software (version 13.0.0.1) was employed for Kα2 stripping and background removal.

### 2.12. In-Vitro Dissolution Studies in the Presence of Electrolytes

The in vitro dissolution studies were carried out in three phases: oral, gastric, and intestinal. For this purpose, electrolyte solutions mimicking the gastrointestinal tract fluids were used. The composition of these electrolytes’ solutions has been mentioned in the Supplementary File. The protocol used has already been described by Minekus et al. [21], and it was modified slightly for the study [22]. The study was carried out for 24 h at 37 °C with continuous stirring. Dry powder equivalent to 25 mg of CIP was added to 4 mL of simulated salivary fluid (SSF) and 25 µL of CaCl_2_(H_2_O)_2_. The pH of the media was modified to 7, followed by the addition of water to bring the volume up to 10 mL. After 3 min, 2 mL of the sample was withdrawn, filtered, and quantified for drug content spectrophotometrically. The withdrawn volume of the sample was replenished with fresh SSF medium. Later, the medium was changed to gastric conditions by adding 8 mL of simulated gastric fluid (SGF) and 5 µL of CaCl_2_(H_2_O)_2_, and 1 M HCl was added dropwise to adjust the pH to 3 to mimic the acidic environment of the stomach, and the volume was adjusted to 20 mL with water. The samples were withdrawn at predetermined time intervals, and the medium was replaced each time to maintain the sink conditions. After 2 h, the medium was modified to mimic the intestinal conditions by adding 16 mL of simulated intestinal fluid (SIF) and 40 µL of CaCl_2_(H_2_O)_2_, followed by maintaining a pH of 7. The final volume was adjusted to 40 mL. The samples were withdrawn at specified time intervals, and the missing volume was replenished with fresh SIF medium. This intestinal phase study was conducted for 24 h.

### 2.13. Cytotoxicity Studies

To assess the impact of our samples on cell viability, we performed the MTT staining technique, originally outlined by Mosmann [23]. The Caco-2 human colon carcinoma cell line (ATCC, Manassas, VA, USA) was seeded onto a 96-well plate at a density of 4 × 10^4^ cells/well in 100 µL of minimal essential medium (MEM) with Earle’s salts, supplemented with 20% heat-inactivated fetal bovine serum (FBS), 2 mmol/L-glutamine, non-essential amino acids, MEM vitamin, sodium pyruvate, and nystatin. Adherent cells were cultured for 24 h at 37 °C with 5% CO_2_ in the aforementioned medium before experimentation.

Subsequently, the medium was aspirated, and the cells were exposed to our samples, which were serially diluted two-fold in MEM. The initial concentration of the samples was 1 mg/mL, with concentrations ranging from 1 mg/mL to 1953 µg/mL. Culture media without samples served as the negative control, while untreated cells were designated as the positive control. Each condition included internal triplicates. The culture plates were then incubated at 37 °C for 24 h. Following the incubation period, 20 µL of MTT solution (thiazolyl blue tetrazolium bromide, Sigma) from a stock solution of 5 mg/mL was added to each well, and the plates were further incubated for 4 h at 37 °C. Following this, 100 µL of SDS solution at a concentration of 10% in 0.01 M HCl was added to each well. After an additional overnight incubation period of 24 h at 37 °C with 5% CO_2_, cell viability was determined by measuring the optical density (OD) at 570/650 nm using an EZ READ 400 ELISA reader (Biochrom, Cambridge, UK). Cell viability was expressed as a percentage of control cells, calculated as described earlier [24]. Statistical analysis was performed using GraphPad Prism 8.0.1 software (GraphPad Software Inc., San Diego, CA, USA).

### 2.14. Anti-Inflammatory Assay

After propagation in Eagle MEM medium, the Caco-2 cells were transferred to 6-well plates at a density of 1 × 10^6^ cells/well. Each sample was used in triplicate. Except for the cell control, 5 µg/mL LPS (Thermo Scientific, Waltham, MA, USA) was added to 30 µg/mL CIP, TC, SA, dry powder with mannitol, and dry powder. Untreated cells served as negative control; LPS-treated cells served as positive control. The plates were then incubated for 24 h at 37 °C in a CO_2_ incubator. The following day, supernatant was aspirated from each well, and the samples were washed with PBS. Subsequently, 1 mL of TRI reagent was added to each well and incubated for 5 min at 4 °C. Finally, the TRI reagent seeded suspension was transferred to 2 mL microcentrifuge tubes and stored at −80 °C until RNA purification.

### 2.15. mRNA Extraction and cDNA Synthesis

Total RNA isolation from the treated and control cells was performed according to the manufacturer’s protocol. Total RNA concentrations and purity were measured using a NanoDrop spectrophotometer (Thermo Scientific, Waltham, MA, USA). First-strand cDNA was synthesized from 2 μg of total RNA using the RevertAid First Strand cDNA Synthesis Kit (Thermo Fisher Scientific Inc., Waltham, MA, USA). Quantitative RT-PCR reactions were carried out using the SensiFAST SYBR^®^ No-ROX Kit (Meridian Bioscience, Newtown, OH, USA), and the validation was performed on a Bio-Rad CFX96 detection system. Primers were designed using the PrimerQuest Tool. The endogenous control was human beta-actin (ACTB).

Human primer sequences used for qPCR were for human Actb sense: 5′-TTCTACAATGAGCTGCGTGTGGCT-3′ and ACTB antisense 5′-TAGCACAGCCTGGATAGCAACGTA-3′; human IL-6 sense: 5′-CAG CTA TGA ACT CCT TCT CCA C-3′ and IL-6 antisense: 5′-GCG GCT ACA TCT TTG GAA TCT-3′; HBD-2 sense: 5′-TTG TCT GAG ATG GCC TCA GGT GGT AAC-3′; HBD-2 antisense: 5′-ATA CTT CAA AAG CAA TTT TCC TTT AT-3′.

### 2.16. In Vitro Anti-Microbial Assay

#### 2.16.1. Inoculum Preparation

The recommendation of the European Committee on Antimicrobial Susceptibility Testing (EUCAST) was followed for the antibacterial studies. Un-supplemented cation-adjusted Mueller–Hinton broth (MH broth) was utilized for the testing of non-fastidious organisms according to the EUCAST standards (Sigma Aldrich, USA). The inoculum preparation involved the use of the direct colony suspension method. The suspensions of the bacteria were standardized to the density of a McFarland of 0.5 using densitometry (Buch-Holm, Herlev, Denmark). The adjusted inoculum was diluted to a concentration of approximately 5 × 10^5^ CFU/mL per tube. Later, 100 µL of the suspension was transferred to sterile 96-well plates (SPL Life Sciences, Miramar, FL, USA), followed by the addition of 64 µg/mL of the drug and samples to be tested in the first well. The dilutions prepared ranged between 32 and 0.064 µg/mL. Each row had one negative control (without bacteria) and a positive control without an agent. The plates were sealed with plastic adhesive closures and incubated at 36 ± 2 °C for 20 h in ambient air to prevent cross-contamination.

#### 2.16.2. Minimum Bactericidal Concentration (MBC)

According to the EUCAST protocol, MBC is the lowest concentration of the test sample that can kill 99.9% of bacteria from the initial bacterial population. In brief, tenfold dilutions of 10 µL from each positive control and sample well (concentration higher than MIC) were applied to the surface of the Mueller–Hinton solid media, following incubation at 36 ± 2 °C. The photographs were taken for the media presenting the countable number of colonies, and then ImageJ software was used to confirm the counts [25]. MBC was calculated using the Forecast function in Microsoft Excel version 2404 (Redmond, WA, USA).

### 2.17. Statistical Analysis

All the experiments were carried out in triplicate and have been mentioned as mean ± SD. *t*-test with one-way analysis of variance (ANOVA) was chosen for statistical analysis, with significant values of <0.05.

## 3. Results and Discussion

### 3.1. Characterization of Polymer

The thiol group was successfully anchored to the backbone of CS, and immobilization of thiol groups on CS was quantified to be 437 ± 24 µM per g of the synthesized polymer. Moreover, the disulfide bonds were quantified to be 98 ± 21 µM in the TC.

### 3.2. Preparation of Dry Powders Following PBD

Here, PBD was used, which is better than the conventional optimization approach because it reduces the time and resources employed. The use of DoE enables a large data set of variables to be tested simultaneously in a few experimental runs. The critical factors were selected by risk assessment tools or literature analysis and entered into the software to generate the experimental runs with a high degree of precision and accuracy. In this work, highly influential parameters were chosen using a seven-factor, two-level, eight-run PBD. Four responses were studied, namely, PDI (Y_1_), particle size (Y_2_), zeta potential (Y_3_), and % EE (Y_4_), against seven independent variables. The acquired experimental runs are presented in Supplementary Table S1.

### 3.3. Influence of the Investigated Parameters on the Dependent Variables

#### 3.3.1. Effect on PDI (Y_1_)

**Figure 1:**
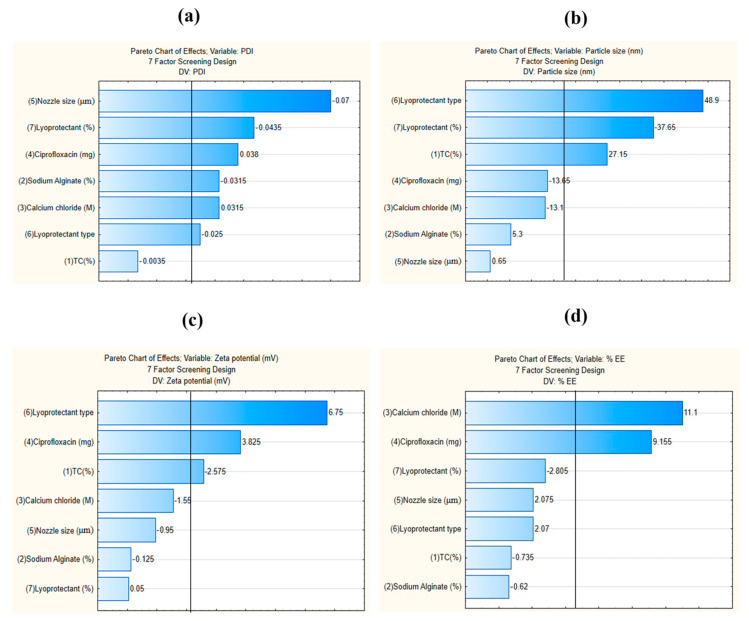
Pareto charts of the effect of the examined independent variables on the outcomes (PDI, particle size, zeta potential, and % EE) of dry powders (**a**–**d**). Bars that exceed the vertical line indicate that the terms are significant (*p* ˂ 0.05).

**Figure 2:**
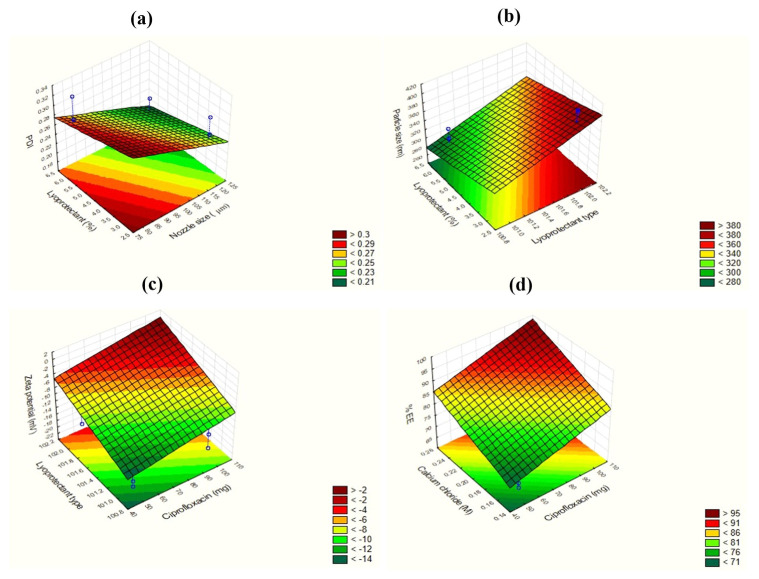
Surface plots obtained by STATISTICA^®^ 12 software presenting the relation between the most influential independent variables on the outcomes (PDI, particle size, zeta potential, and % EE) of dry powders (**a**–**d**).

The PDI values ranged from 0.178 to 0.324 based on the factors. In this case, all the independent factors except the concentration of polymer TC were found to influence the outcome of PDI (Figure 1a); however, the most significant ones were the nozzle size of the encapsulator and the % of cryoprotectant used (Figure 2a).

The established linear relationship for the estimation of Y_1_ was found to be

Y_1_= 0.271 − 0.003X_1_ − 0.03X_2_ + 0.03X_3_ + 0.03X_4_ − 0.07X_5_ − 0.03X_6_ − 0.04X_7_

As the nozzle size decreased from 120 µm to 80 µm, the PDI was significantly influenced. Also, the nature of the excipient used as lyoprotectant affected the PDI of the particles. Mannitol was less prone to hygroscopicity and therefore maintained good powder characteristics compared to the use of trehalose. The use of mannitol therefore improved powder redispersibility and hence the PDI and morphology [26]. It can therefore be hypothesized that the use of mannitol during lyophilization and low nozzle size during encapsulation can be considered optimal factors during future nanoparticulate preparation.

#### 3.3.2. Effect on Particle Size (Y_2_)

The average particle size of the nanobead-based powders varied between 304.1 nm and 403.5 nm. As can be seen in Figure 1b, the most influential independent variables for size were found to be the type of lyoprotectant and % of lyoprotectant (Figure 2b). As the % of lyoprotectant increased, the particle size decreased. This is due to the reason that cryoprotectant prevents the aggregation of particles during the process of drying and, therefore, the final particle size outcome [27]. Likewise, the aggregation of the samples was also found to be lower for the samples containing mannitol than those containing trehalose. Moreover, it has been stated that the use of mannitol also shortens the reconstitution time of the powders compared to trehalose [28]. The established linear relationship for the estimation of Y_2_ is as follows:

Y_2_ = 344.6 + 27.15X_1_ + 5.3X_2_ − 13.1X_3_ − 13.6X_4_ + 0.65X_5_ + 48.9X_6_ − 37.65X_7_

#### 3.3.3. Effect on Zeta Potential (Y_3_)

The zeta potential varied between −10.7 mV and 22.3 mV. The established relationship for the estimation of Y_3_ is as follows:

Y_3_ = −17 − 2.57X_1_ − 0.125X_2_ − 1.55X_3_ + 3.82X_4_ − 0.95X_5_ + 6.75X_6_ − 0.05X_7_

The change in zeta potential was influenced highly by the type of lyoprotectant used and the concentration of the drug (Figure 1c). The use of mannitol improved the morphology of the particles, due to which the particles remained segregated, and hence, the zeta potential was found to be better due to good stability [29]. The high concentration of the encapsulated drug also affects the surface charge on the nanoparticles due to the high integrity of the structure compared to the hollow or blank nanocarriers [30]. Figure 2c also shows the relation between the drug concentration and the type of lyoprotectant in a surface plot.

#### 3.3.4. Effect on %EE (Y_4_)

As can be seen in Figure 1d, the increase in the concentration of the drug and crosslinker improved the % EE. The established relationship for the estimation of Y_4_ is as follows:

Y_4_ = 78.7 − 0.735X_1_ − 0.62X_2_ + 11X_3_ + 9.15X_4_ + 2.075X_5_ + 2.07X_6_ − 2.805X_7_

Figure 2d (surface plot) also establishes the relationship between the drug and cross-linker in increasing the % EE. The high ratio of the cross-linker enables electrostatic interaction between the polymers and therefore allows for better entrapment of the drug within the solution. These electrostatic interactions also prevent premature leakage of the entrapped drug unless a suitable medium comes in contact to leach out the drug.

### 3.4. Morphological Examination

**Figure 3:**
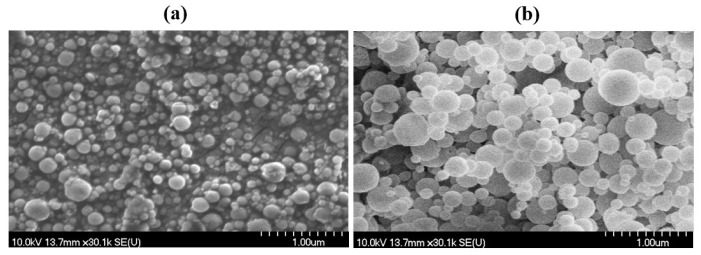
SEM micrographs of dry powder with mannitol (**a**) and dry powder with trehalose (**b**).

The SEM images present the smooth morphology of the mannitol-based dry powders (Figure 3a), with particles homogenously distributed and narrow sizes. On the contrary, the dry powders with trehalose exhibit patches of agglomeration with large particles, as can be seen in Figure 3b.

### 3.5. Thermal and Structural Investigation

**Figure 4:**
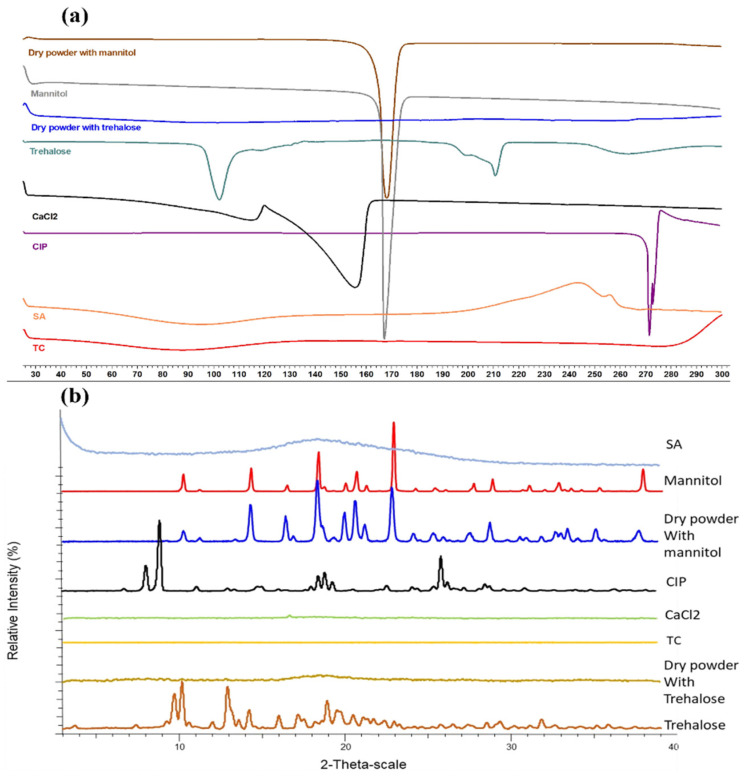
DSC curves of the polymer, excipients, drug, and dry powder samples, exo ↑ (**a**), and XRPD patterns of the polymer, excipients, drug, and dry powder samples (**b**).

The DSC curves present the thermal behavior of the excipients, drug, and dry powders. As can be seen in Figure 4a, CIP displays a sharp endotherm at 272.58 °C [31], which is the melting peak; however, it is absent in the dry powder samples presenting the encapsulation of the drug within the particles in amorphous form. The lyoprotectant, mannitol, demonstrated a melting endotherm at 170.11 °C, which was also present in the dry powder mannitol sample, showing its recrystallization behavior during the cooling of the samples [32]. Trehalose dihydrate thermograms present the order-to-disorder phase transition with the first endotherm at 100.12 °C, corresponding to the dehydration/loss of unbound water. Furthermore, another endotherm at 212.03 °C displays the fusion of anhydrous β form of trehalose [33]. However, these peaks were absent in the dry samples with trehalose, showing the amorphous nature of trehalose upon drying. CaCl_2_, which was used as a cross-linker, presented a melting peak at 156 °C [34], which was, however, not present in the dry powder samples, showing the stability of the excipient after drying. TC and SA did not exhibit any sharp peaks individually or in the dry samples.

The XRPD patterns provided information about the structural changes in the polymer, excipients, drug, and dry samples. As can be seen in Figure 4b, the crystalline nature of CIP was not dominant in the dry powder samples, highlighting the change in the crystalline nature of CIP to amorphous on loading. Similarly, crystalline trehalose was found to be completely amorphous upon drying, with no distinct peaks in the dry samples. However, the distinct crystalline peaks of mannitol were found to be present in the dry powder samples as well, which correlated well with the DSC curves.

### 3.6. In Vitro Dissolution Studies in the Presence of Electrolytes

**Figure 5:**
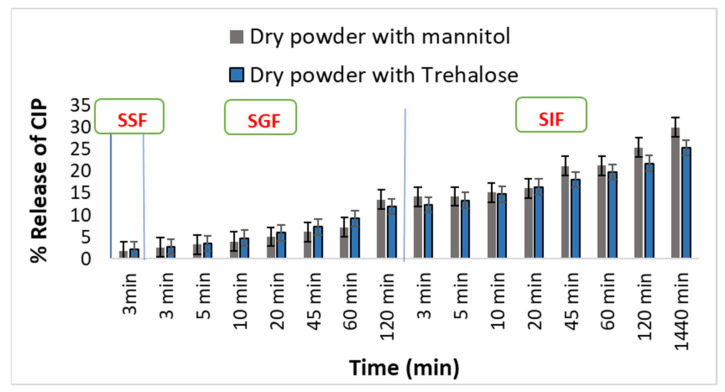
% Release of CIP from dry powders in simulated saliva fluid (SSF), simulated gastric fluid (SGF), and simulated intestinal fluid (SIF).

The in vitro dissolution studies were conducted under three different gastrointestinal conditions by altering the media to SSF, SGF, and SIF (Figure 5). The study provided insight into the release pattern of the dry powders from the point of contact with saliva to the intestinal fluids. Dry powder with mannitol presented a low drug release in SSF (i.e., 1.72%). However, the drug release from mannitol-based dry powder and trehalose-based dry powder remained almost the same in SGF. On the other hand, the dry powder with trehalose showed the release in a controlled manner in the SIF, with up to 25% drug release. Nonetheless, both the dry powder samples exhibited good results and can be further evaluated for permeation across the gut epithelial cell line.

### 3.7. Cytotoxicity Studies

**Figure 6:**
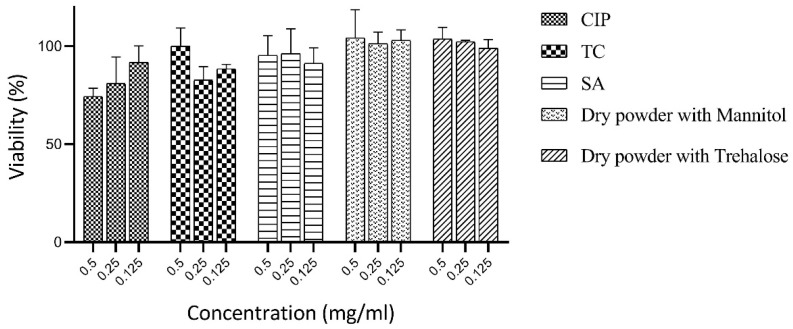
Cell viability MTT assay on Caco-2 cells, with samples concentrations of 0.125, 0.25, and 0.5 mg/mL. Untreated cells served as a control, with 100% viability.

The cell viability assay was performed using the MTT method at three varying concentrations of the samples and drug, i.e., 0.125, 0.25, and 0.5 mg/mL, on the Caco-2 cells (Figure 6). Untreated cells served as a control, with 100% viability. The drug, polymers, and dry samples exhibited promising cell viability, presenting the biocompatible nature of the ingredients. Low toxicity and high biocompatibility are directly associated and are highly essential to human safety; hence, it can be concluded that the samples prepared herein can be further exploited in animal studies in the future.

### 3.8. Anti-Inflammatory Assay

In this study, we explored the anti-inflammatory properties of our samples for innate immune responses, focusing on a cytokine, IL-6, and antimicrobial peptide, human beta defensin-2 (HBD-2) production. IL-6 is recognized for its pivotal role in driving the differentiation of T helper 17 (Th17) cells, which exacerbate inflammatory reactions observed in Crohn’s disease (CD) and ulcerative colitis (UC).

**Figure 7:**
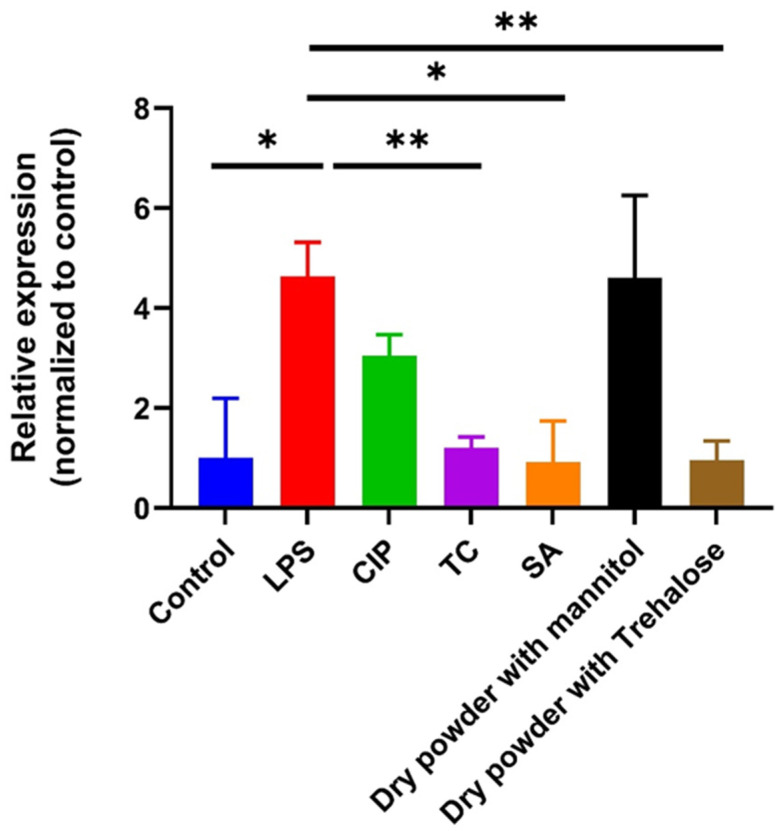
Relative expression of IL-6 on Caco-2 cells; untreated cells served as control. Results are expressed as mean ± SD (*n* = 3 independent measurements). Level of significance: (* *p* < 0.05), (** *p* < 0.01).

Moreover, the antiapoptotic signals mediated by the IL-6/IL-6 receptor (IL-6R) axis contribute to the persistence of Th1 cells at inflamed sites. Recent evidence suggests that fecal bacteria from both the active and remission phases of UC provoke excessive IL-6 production [35]. Given the importance of IL-6 signaling, we evaluated the gene expression levels of IL-6 upon stimulation with LPS and our samples. Our findings revealed that the samples significantly attenuated LPS-induced IL-6 expression compared to cells treated with LPS alone (Figure 7). These results suggest the potential role of our develped samples in mitigating inflammation in the human gut.

**Figure 8:**
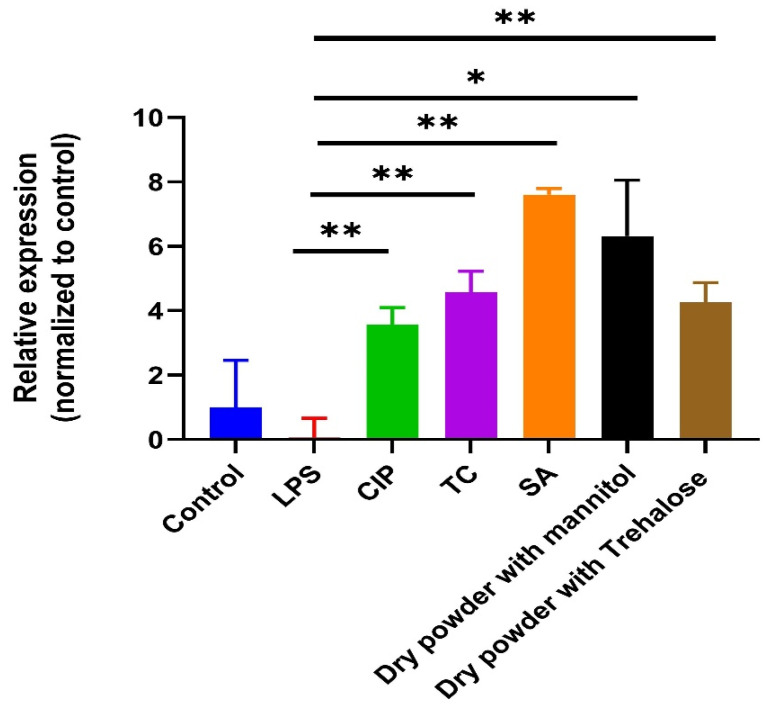
Relative expression of HBD-2 on Caco-2 cells; untreated cells served as control. Results are expressed as mean ± SD (*n* = 3 independent measurements). Level of significance: (* *p* < 0.05), (** *p* < 0.01).

The Caco-2 cell line, known for its high production of HBD-2, serves a protective function on organ surfaces exposed to microorganisms. HBD-2 exhibits antimicrobial activity against various pathogens, particularly Gram-negative bacteria and yeasts [36,37]. Notably, HBD-2 is inducible by inflammatory stimuli, prompting an immediate antimicrobial response by epithelial cells upon encountering microorganisms or injury. This innate defense mechanism is distinct from leukocyte-dependent immune responses, underscoring the significance of HBD-2 in human epithelial defense [38]. We assessed the effects of our substances on LPS-induced antimicrobial HBD-2 production and found that all our samples significantly enhanced HBD-2 production compared to the LPS control, suggesting an augmenting effect of our samples on antimicrobial peptide production (Figure 8).

### 3.9. Antibacterial Assay

**Figure 9:**
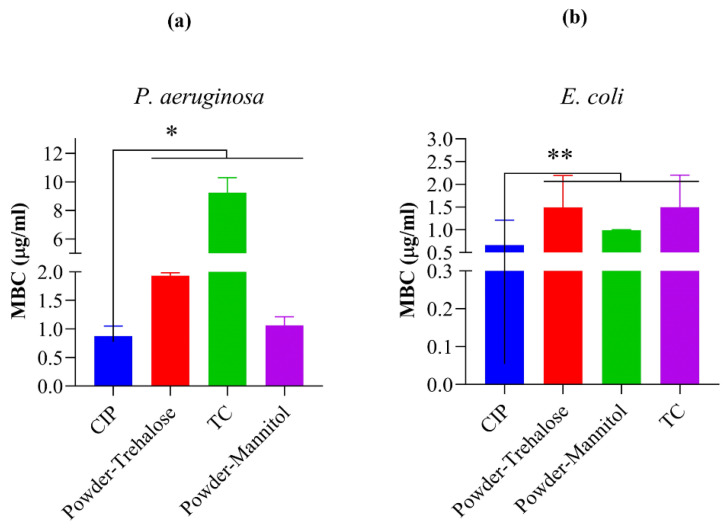
MBC values of the samples in µg/mL for *Pseudomonas aeruginosa* (ATCC^®^ 27853) (**a**) and *Escherichia coli* (ATCC^®^ 25922) (**b**). Results are expressed as mean ± SD (*n* = 3 independent measurements). Level of significance: (* *p* < 0.05), (** *p* < 0.01).

*P. aeruginosa* and *E. coli* were used for the antibacterial assay to determine MBC. *P. aeruginosa* is a Gram-positive bacterium responsible for urinary tract and respiratory diseases, whereas *E. coli* is a Gram-negative anaerobic bacterium that causes stomach infections. As our research was aimed at targeting gastrointestinal ailments using oral delivery, these bacteria models were found to be relevant to the work. The MBC study was carried out for the nascent drug, dry powders, and TC (Figure 9). As TC is mentioned to have some antibacterial properties, this fact was also explored. The MBC values for the dry powder samples with mannitol were found to be lower, i.e., 1.06 µg/mL and 0.99 µg/mL for *P. aeruginosa* and *E. coli*, respectively, compared to the dry powder samples with trehalose (1.93 µg/mL for *P. aeruginosa* and 1.49 µg/mL for *E. coli*). Moreover, the polymer TC also exhibited bactericidal activity with high MBC in the case of *P. aeruginosa*, i.e., 9.25 µg/mL, but in the case of *E. coli*, MBC was found to be 1.49 µg/mL. Altogether, the results were quite promising, as the powder samples exhibited an MBC almost similar to that of CIP.

## 4. Conclusions

Oral delivery remains the most exploited route of administration for antibiotics, and therefore this study provided valuable insight into the scope of employing mucoadhesive polymers such as TC along with SA, which can improve the residence time of the encapsulated antibiotic in the gastrointestinal tract. The polymers are not only biocompatible but also biodegradable, which are the main human health and ethical concerns. The use of the B-390 encapsulator for encapsulation of the active agent proved to be the simplest and most easily scalable approach for the fabrication of a nanosystem. This work showed that SA can not only act as a carrier but also impart anti-inflammatory characteristics that were demonstrated by the anti-inflammatory studies on Caco-2 cells. Furthermore, the antibacterial activity of the prepared samples was promising and provided a pathway for future animal studies. Altogether, the use of DoE allowed for the holistic screening and optimization of the critical process and material parameters to allow for better outcomes compared to traditional optimization approaches.

## Figures and Tables

**Table 1:** Experimental factors and their levels.

Factor	Factor Significance	Level (−1)	Level (+1)
X_1_	TC %	0.5	1
X_2_	SA %	1	1.5
X_3_	CaCl_2_ (M)	0.15	0.25
X_4_	CIP (mg)	50	100
X_5_	Nozzle size (µm)	80	120
X_6_	Lyoprotectant type	Mannitol	Trehalose
X_7_	Lyoprotectant %	3	6

**Table 2:** The input factor levels in a 7-factor, 2-level, 8-run PBD.

Run Code	TC %	SA (%)	CaCl_2_ (M)	CIP (mg)	Nozzle Size (µm)	Lyoprotectant Type	Lyoprotectant %
1	0.5	1	0.15	100	120	Mannitol	3
2	0.5	1	0.25	100	80	Trehalose	6
3	0.5	1.5	0.15	50	120	Mannitol	6
4	0.5	1.5	0.25	50	80	Trehalose	3
5	1	1	0.15	50	80	Mannitol	6
6	1	1	0.25	50	120	Trehalose	3
7	1	1.5	0.15	100	80	Trehalose	3
8	1	1.5	0.25	100	120	Mannitol	6

## Data Availability

Data are contained within the article and Supplementary Materials.

## Supplementary Materials

The following supporting information can be downloaded at: https://www.mdpi.com/article/10.3390/pharmaceutics16060691/s1, Table S1: Experimental runs and their outcomes; Table S2: % *w*/*w* Drug content; Figure S1: Predicted vs. observed values PDI; Figure S2: Predicted vs. observed values particle size; Figure S3: Predicted vs. observed values zeta potential; Figure S4: Predicted vs. observed values % EE; Table S3: Volumes required for the preparation of SSF, SGF and SIF.

## References

[1] Roberts C.A., Buikstra J.E. (2019). Bacterial infections. Ortner’s Identification of Pathological Conditions in Human Skeletal Remains.

[2] Abban M.K., Ayerakwa E.A., Mosi L., Isawumi A. (2023). The burden of hospital acquired infections and antimicrobial resistance. Heliyon.

[3] Raza A., Sime F.B., Cabot P.J., Maqbool F., Roberts J.A., Falconer J.R. (2019). Solid nanoparticles for oral antimicrobial drug delivery: A review. Drug Discov. Today.

[4] Sathish D., Himabindu S., Shravan Kumar Y., Madhusudan Rao Y. (2011). Floating drug delivery systems for prolonging gastric residence time: A review. Curr. Drug Deliv..

[5] de Oliveira Cardoso V.M., Gremião M.P.D., Cury B.S.F. (2020). Mucin-polysaccharide interactions: A rheological approach to evaluate the effect of pH on the mucoadhesive properties. Int. J. Biol. Macromol..

[6] Agüero L., Zaldivar-Silva D., Peña L., Dias M.L. (2017). Alginate microparticles as oral colon drug delivery device: A review. Carbohydr. Polym..

[7] Mirshafiey A., Khodadadi A., Rehm B., Khorramizadeh M., Eslami M., Razavi A., Saadat F. (2005). Sodium alginate as a novel therapeutic option in experimental colitis. Scand. J. Immunol..

[8] Anitha A., Deepagan V., Rani V.D., Menon D., Nair S., Jayakumar R. (2011). Preparation, characterization, in vitro drug release and biological studies of curcumin loaded dextran sulphate–chitosan nanoparticles. Carbohydr. Polym..

[9] Egorov A.R., Kurliuk A.V., Rubanik V.V., Kirichuk A.A., Khubiev O., Golubev R., Lobanov N.N., Tskhovrebov A.G., Kritchenkov A.S. (2022). Chitosan-Based Ciprofloxacin Extended Release Systems: Combined Synthetic and Pharmacological (In Vitro and In Vivo) Studies. Molecules.

[10] Egorov A.R., Kurasova M.N., Khubiev O., Bogdanov N.A., Tskhovrebov A.G., Kirichuk A.A., Khrustalev V.N., Rubanik V.V., Rubanik V.V., Kritchenkov A.S. (2022). Ciprofloxacin chitosan conjugate: Combined antibacterial effect and low toxicity. Mendeleev Commun..

[11] Anitha A., Deepa N., Chennazhi K., Nair S., Tamura H., Jayakumar R. (2011). Development of mucoadhesive thiolated chitosan nanoparticles for biomedical applications. Carbohydr. Polym..

[12] Egorov A.R., Kirichuk A.A., Rubanik V.V., Rubanik V.V., Tskhovrebov A.G., Kritchenkov A.S. (2023). Chitosan and Its Derivatives: Preparation and Antibacterial Properties. Materials.

[13] Shrivastava P., Vyas S., Sharma R., Mody N., Gautam L., Jain A., Vishwakarma N., Vyas S.P. (2020). Nanotechnology for oral drug delivery and targeting. Nanoengineered Biomaterials for Advanced Drug Delivery.

[14] Gedawy A., Dass C.R., Al-Salami H. (2020). Polydimethylsiloxane-customized nanoplatform for delivery of antidiabetic drugs. Ther. Deliv..

[15] Mukhtar M., Pallagi E., Csóka I., Benke E., Farkas Á., Zeeshan M., Burián K., Kókai D., Ambrus R. (2020). Aerodynamic properties and in silico deposition of isoniazid loaded chitosan/thiolated chitosan and hyaluronic acid hybrid nanoplex DPIs as a potential TB treatment. Int. J. Biol. Macromol..

[16] Kast C.E., Bernkop-Schnürch A. (2001). Thiolated polymers—Thiomers: Development and in vitro evaluation of chitosan–thioglycolic acid conjugates. Biomaterials.

[17] Mukhtar M., Zesshan M., Khan S., Shahnaz G., Khan S.A., Sarwar H.S., Pasha R.A., Ali H. (2020). Fabrication and optimization of pH-sensitive mannose-anchored nano-vehicle as a promising approach for macrophage uptake. Appl. Nanosci..

[18] Sohail M.F., Javed I., Hussain S.Z., Sarwar S., Akhtar S., Nadhman A., Batool S., Bukhari N.I., Saleem R.S.Z., Hussain I. (2016). Folate grafted thiolated chitosan enveloped nanoliposomes with enhanced oral bioavailability and anticancer activity of docetaxel. J. Mater. Chem. B.

[19] Rahman Z., Zidan A.S., Habib M.J., Khan M.A. (2010). Understanding the quality of protein loaded PLGA nanoparticles variability by Plackett–Burman design. Int. J. Pharm..

[20] Cho A.R., Chun Y.G., Kim B.K., Park D.J. (2014). Preparation of alginate–CaCl_2_ microspheres as resveratrol carriers. J. Mater. Sci..

[21] Minekus M., Alminger M., Alvito P., Ballance S., Bohn T., Bourlieu C., Carrière F., Boutrou R., Corredig M., Dupont D. (2014). A standardised static in vitro digestion method suitable for food–an international consensus. Food Funct..

[22] Martinović J., Lukinac J., Jukić M., Ambrus R., Planinić M., Šelo G., Klarić A.-M., Perković G., Bucić-Kojić A. (2023). Physicochemical characterization and evaluation of gastrointestinal in vitro behavior of alginate-based microbeads with encapsulated grape pomace extracts. Pharmaceutics.

[23] Mosmann T. (1983). Rapid colorimetric assay for cellular growth and survival: Application to proliferation and cytotoxicity assays. J. Immunol. Methods.

[24] Banat H., Csóka I., Paróczai D., Burian K., Farkas Á., Ambrus R. (2024). A Novel Combined Dry Powder Inhaler Comprising Nanosized Ketoprofen-Embedded Mannitol-Coated Microparticles for Pulmonary Inflammations: Development, In Vitro–In Silico Characterization, and Cell Line Evaluation. Pharmaceuticals.

[25] Huang C., Becker M.F., Keto J.W., Kovar D. (2007). Annealing of nanostructured silver films produced by supersonic deposition of nanoparticles. J. Appl. Phys..

[26] Torge A., Grützmacher P., Mücklich F., Schneider M. (2017). The influence of mannitol on morphology and disintegration of spray-dried nano-embedded microparticles. Eur. J. Pharm. Sci..

[27] Almalik A., Alradwan I., Kalam M.A., Alshamsan A. (2017). Effect of cryoprotection on particle size stability and preservation of chitosan nanoparticles with and without hyaluronate or alginate coating. Saudi Pharm. J..

[28] Luo W.-C., Beringhs A.O.R., Kim R., Zhang W., Patel S.M., Bogner R.H., Lu X. (2021). Impact of formulation on the quality and stability of freeze-dried nanoparticles. Eur. J. Pharm. Biopharm..

[29] Larsson M., Hill A., Duffy J. (2012). Suspension stability; why particle size, zeta potential and rheology are important. Annu. Trans. Nord. Rheol. Soc..

[30] Fatouros D.G., Antimisiaris S.G. (2002). Effect of amphiphilic drugs on the stability and zeta-potential of their liposome formulations: A study with prednisolone, diazepam, and griseofulvin. J. Colloid Interface Sci..

[31] Shazly G.A. (2017). Ciprofloxacin controlled-solid lipid nanoparticles: Characterization, in vitro release, and antibacterial activity assessment. BioMed Res. Int..

[32] Ambrus R., Csóka I., Fenyes E., Orosz L., Sarkadi Á.N., Burián K., Kókai D., Mukhtar M. (2024). Holistic study design following Quality by Design approach for fabrication of hybrid polymeric nanoparticulate based dry powders as carriers for Ciprofloxacin. J. Pharm. Sci..

[33] Li X., Mansour H.M. (2011). Physicochemical characterization and water vapor sorption of organic solution advanced spray-dried inhalable trehalose microparticles and nanoparticles for targeted dry powder pulmonary inhalation delivery. Aaps Pharmscitech.

[34] Iswandana R., Putri K.S.S., Wulandari F.R., Najuda G., Sari S.P., Djajadisastra J. (2018). Preparation of calcium alginate-tetrandrine beads using ionic gelation method as colon-targeted dosage form. J. Appl. Pharm. Sci..

[35] Shahini A., Shahini A. (2023). Role of interleukin-6-mediated inflammation in the pathogenesis of inflammatory bowel disease: Focus on the available therapeutic approaches and gut microbiome. J. Cell Commun. Signal..

[36] Fusco A., Savio V., Perfetto B., Mattina R., Donnarumma G. (2022). Antimicrobial peptide human β-defensin-2 improves in vitro cellular viability and reduces pro-inflammatory effects induced by enteroinvasive Escherichia coli in Caco-2 cells by inhibiting invasion and virulence factors’ expression. Front. Cell. Infect. Microbiol..

[37] Gácser A., Tiszlavicz Z., Németh T., Seprényi G., Mándi Y. (2014). Induction of human defensins by intestinal Caco-2 cells after interactions with opportunistic Candida species. Microbes Infect..

[38] Schröder J.-M., Harder J. (1999). Human beta-defensin-2. Int. J. Biochem. Cell Biol..

