# Supplementary material for: Fabrication of Ciprofloxacin-Loaded Sodium Alginate Nanobeads Coated with Thiol-Anchored Chitosan Using B-390 Encapsulator Following Optimization by DoE
Source: Pharmaceutics. 2024 May 21;16(6):691. doi: 10.3390/pharmaceutics16060691 (PMC11206434; doi:10.3390/pharmaceutics16060691)
Supplement: Supplementary file 1 [file pharmaceutics-16-00691-s001.zip › pharmaceutics-3014271-supplementary.pdf]

Fabrication of Ciprofloxacin-Loaded Sodium Alginate Nanobeads Coated with Thiol-Anchored Chitosan Using B-390 Encapsulator Following Optimization by DoE

Mahwash Mukhtar [1], Ildikó Csóka [1], Josipa Martinović [2], Gordana Šelo [2], Ana Bucić-Kojić [2], László Orosz [3], Dóra Paróczai [3], Katalin Burian [3] and Rita Ambrus [1,*]

[1] Institute of Pharmaceutical Technology and Regulatory Affairs, Faculty of Pharmacy, University of Szeged, Eötvös u.6, 6720 Szeged, Hungary; mahwash.mukhtar@szte.hu (M.M.); csoka.ildiko@szte.hu (I.C.)

[2] Faculty of Food Technology Osijek, Josip Juraj Strossmayer University of Osijek, F. Kuhača 18, 31 000 Osijek, Croatia; jgrgic2@ptfos.hr (J.M.); abucic@ptfos.hr (A.B.-K.)

[3] Department of Medical Microbiology, Faculty of Medicine, University of Szeged, Dóm Square 10, 6720 Szeged, Hungary; orosz.laszlo@med.u-szeged.hu (L.O.); paroczai.dora@med.u-szeged.hu (D.P.); burian.katalin@med.u-szeged.hu (K.B.)

* Correspondence: ambrus.rita@szte.hu

Table S1: Experimental runs and their outcomes

Standard Run	TC(%)	Sodium Alginate (%)	Calcium chloride (M)	Ciprofloxacin (mg)	Nozzle size (μm)	Lyoprotectant type	Lyoprotectant (%)	PDI	Particle size (nm)	Zeta potential (mV)	% EE
	Design: 2**(7-4) design (|No active dataset|)										
1	0.5	1.0	0.15	100	120	Mannitol	3	0.291	322.8	-16.9	79.9
2	0.5	1.0	0.25	100	80	Trehalose	6	0.324	320.3	-10.7	88.1
3	0.5	1.5	0.15	50	120	Mannitol	6	0.178	304.1	-20.8	67.3
4	0.5	1.5	0.25	50	80	Trehalose	3	0.298	376.9	-14.7	81.1
5	1.0	1.0	0.15	50	80	Mannitol	6	0.276	325.3	-22.3	65.1
6	1.0	1.0	0.25	50	120	Trehalose	3	0.256	399.4	-18.1	83.0
7	1.0	1.5	0.15	100	80	Trehalose	3	0.301	403.5	-11.9	78.5
8	1.0	1.5	0.25	100	120	Mannitol	6	0.244	304.5	-21.1	86.7

Table S2: % w/w Drug content

Samples	Drug content (% w/w)
1	1.285
2	1.153
3	1.110
4	1.431
5	1.014
6	1.032
7	1.463
8	1.504

Figure S1: Predicted vs. observed values PDI

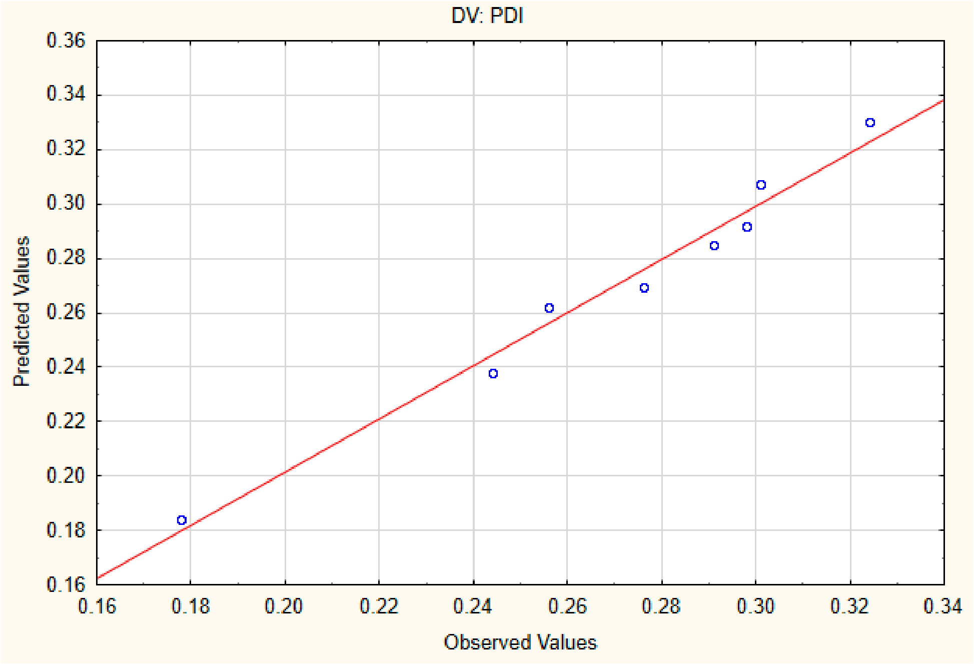

Figure S2: Predicted vs. observed values particle size

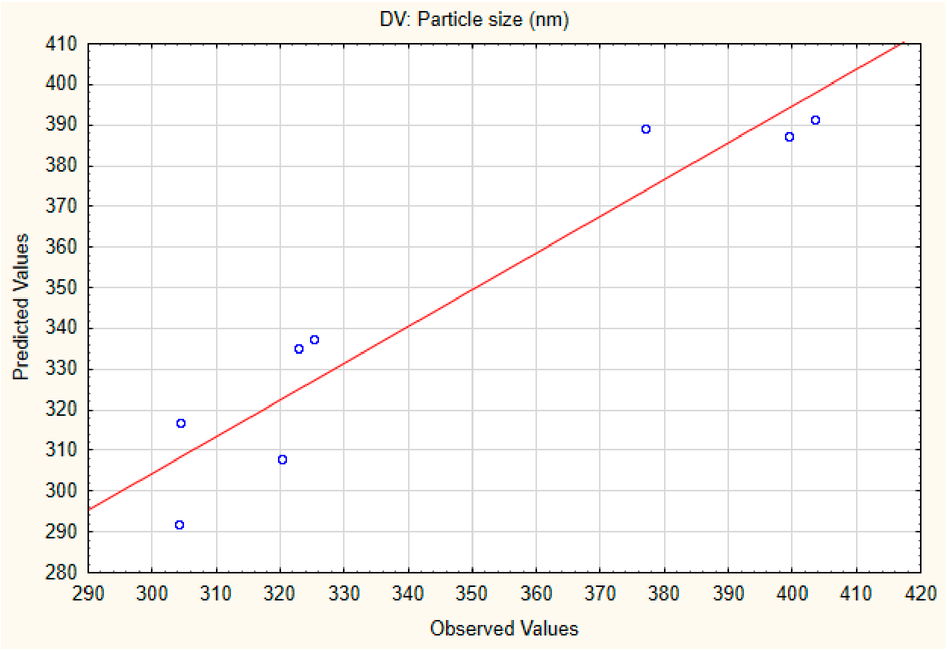

Figure S3: Predicted vs. observed values zeta potential

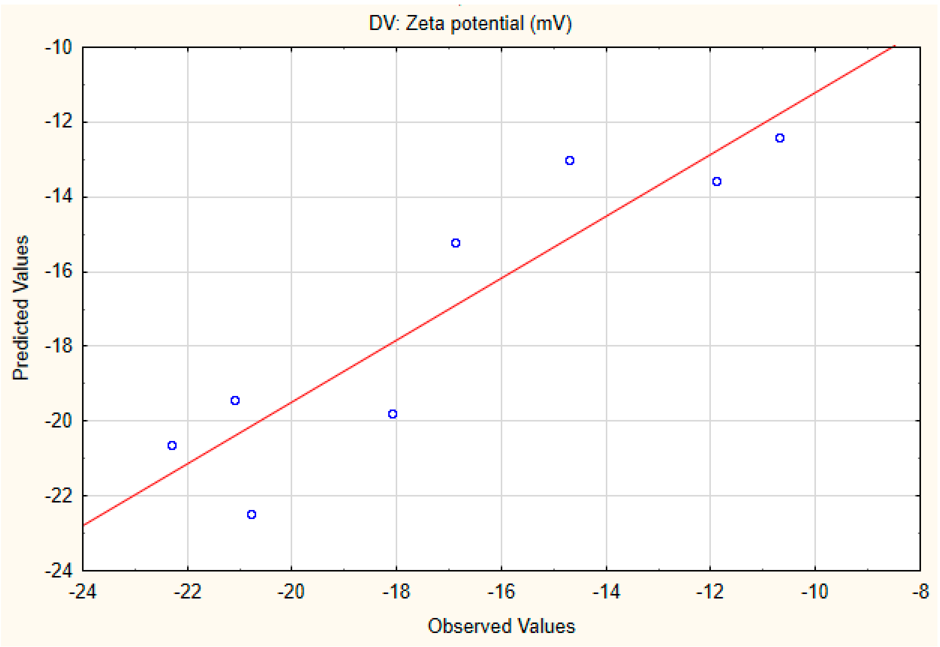

Figure S4: Predicted vs. observed values % EE

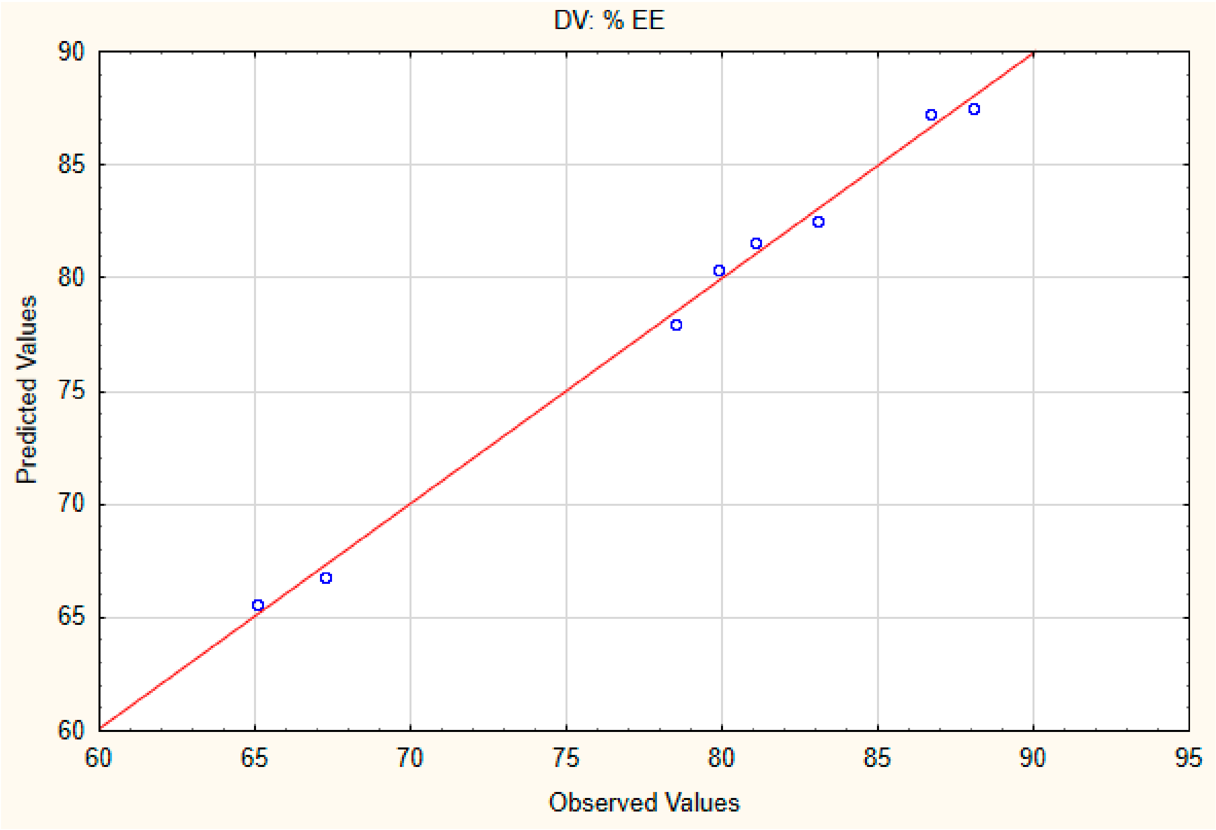

Table S3: Volumes required for the preparation of SSF, SGF and SIF

V (ml)	SSF	SGF	SIF
**KCl**	15.1	6.9	6.8
**KH_2PO_4**	3.7	0.9	0.8
**$NaHCO_3$**	6.8	25	42.5
**NaCl**	-	47.2	9.6
**$MgCl_2(H_2O)6$**	0.5	0.1	1.1
**$(NH_4)_2CO_3$**	0.06	0.5	-
**H_2O**	373.84	319.4	339.2
